# Phybers: a package for brain tractography analysis

**DOI:** 10.3389/fnins.2024.1333243

**Published:** 2024-03-11

**Authors:** Lazara Liset González Rodríguez, Ignacio Osorio, Alejandro Cofre G., Hernan Hernandez Larzabal, Claudio Román, Cyril Poupon, Jean-François Mangin, Cecilia Hernández, Pamela Guevara

**Affiliations:** ^1^Department of Electrical Engineering, Faculty of Engineering, Universidad de Concepción, Concepción, Chile; ^2^Latin American Brain Health Institute (BrainLat), Universidad Adolfo Ibañez, Santiago, Chile; ^3^Centro de Investigación y Desarrollo en Ingeniería en Salud, Universidad de Valparaíso, Valparaíso, Chile; ^4^CEA, CNRS, Baobab, Neurospin, Université Paris-Saclay, Gif-sur-Yvette, France; ^5^Department of Computer Science, Faculty of Engineering, Universidad de Concepción, Concepción, Chile; ^6^Center for Biotechnology and Bioengineering (CeBiB), Santiago, Chile

**Keywords:** diffusion MRI, tractography, python, white matter segmentation, fiber clustering, bundle atlas

## Abstract

We present a Python library (Phybers) for analyzing brain tractography data. Tractography datasets contain streamlines (also called fibers) composed of 3D points representing the main white matter pathways. Several algorithms have been proposed to analyze this data, including clustering, segmentation, and visualization methods. The manipulation of tractography data is not straightforward due to the geometrical complexity of the streamlines, the file format, and the size of the datasets, which may contain millions of fibers. Hence, we collected and structured state-of-the-art methods for the analysis of tractography and packed them into a Python library, to integrate and share tools for tractography analysis. Due to the high computational requirements, the most demanding modules were implemented in C/C++. Available functions include brain Bundle Segmentation (FiberSeg), Hierarchical Fiber Clustering (HClust), Fast Fiber Clustering (FFClust), normalization to a reference coordinate system, fiber sampling, calculation of intersection between sets of brain fibers, tools for cluster filtering, calculation of measures from clusters, and fiber visualization. The library tools were structured into four principal modules: Segmentation, Clustering, Utils, and Visualization (Fibervis). Phybers is freely available on a GitHub repository under the GNU public license for non-commercial use and open-source development, which provides sample data and extensive documentation. In addition, the library can be easily installed on both Windows and Ubuntu operating systems through the *pip* library.

## 1 Introduction

Structural brain connectivity can be studied using Diffusion Magnetic Resonance Imaging (dMRI), (Le Bihan and Breton, 1985). This is a non-invasive, in vivo technique that provides microscopic-scale information on brain white matter (WM) by measuring the movement of water molecules in brain tissues. Using diffusion local model reconstruction (Basser et al., 1994; Tuch, 2004; Wedeen et al., 2005; Tournier et al., 2007; Yeh et al., 2010; Fick et al., 2019) and tractography algorithms on dMRI data (Basser et al., 2000; Malcolm et al., 2010; Smith et al., 2012; Tournier et al., 2012; Wasserthal et al., 2019), it is possible to calculate the main trajectory of 3-dimensional (3D) WM fascicles as a set of 3D polylines. These sets of streamlines, for simplicity, are also known as “fibers” even though they do not represent single axons.

Over the years, the tools for analyzing tractography datasets have evolved along with improvements in MRI equipment and reconstruction, and tractography algorithms. Today, brain tractography data are quite complex, containing long and short fibers, as well as noise, and intricate geometrical configurations. In addition, these datasets can contain several million fibers for probabilistic tractography, yielding additional computational requirements, especially when performing multi-subject analysis. This is why there are numerous tractography data analysis algorithms that seek to cluster (O'Donnell et al., 2006; Garyfallidis et al., 2012; Siless et al., 2018; Vázquez et al., 2020; Chen et al., 2023), identify patterns (Guevara et al., 2012, 2017; Kumar and Desrosiers, 2016; Román et al., 2017), segment (Donnell and Westin, 2007; Wassermann et al., 2016; Labra et al., 2017; Garyfallidis et al., 2018; Wasserthal et al., 2018; Zhang et al., 2020a; Vindas et al., 2023), filter (Garyfallidis et al., 2014; Mendoza et al., 2021), visualize (Wang et al., 2007; Riviére et al., 2011; Garyfallidis et al., 2014; Chamberland et al., 2015; Norton et al., 2017; Tournier et al., 2019; Zhang et al., 2020b; Franke et al., 2021), and calculate measures on these data (Yeh et al., 2013; Garyfallidis et al., 2014). Due to the complexity of tractography data, the algorithms are usually difficult to use and require a deep understanding of the file formats, input parameters, and results. Hence, to simplify and promote its use, several groups have created and distributed software packages for the processing of dMRI images. Such tools include algorithms for different stages of the dMRI processing pipeline, from image distortion correction to tractography analysis. The final goal is to have methods for the processing of tractography data, for a better description of WM fibers based on high-quality data (Zhang et al., 2018; Radwan et al., 2022; Román et al., 2022) and the study of WM microstructure on healthy subjects (Lebel et al., 2019; Li et al., 2020; Schilling et al., 2021; Zekelman et al., 2022) and pathological brains (O'Donnell et al., 2017; Zhao et al., 2017; Goldsmith et al., 2018; Mito et al., 2018; Roy et al., 2020; Buyukturkoglu et al., 2022).

There are a wide variety of tools available for the processing of dMRI data. Tables 1, 2 summarize and describe the main software packages used by the medical imaging research community. The table lists the main features and functionalities of the tools, such as programming language, operating system (OS), distribution license, dMRI format, tractography format, diffusion-weighted (DW) model reconstruction, fiber tracking, fiber clustering, bundle segmentation, visualization, and calculation of fiber measures. The software considered are: BrainSUITE (Shattuck and Leahy, 2002), Camino (Cook et al., 2006), Diffusion toolkit (Wang et al., 2007), ExploreDTI (Leemans et al., 2009), FSL (Smith et al., 2004; Woolrich et al., 2009; Jenkinson et al., 2012), MRtrix (Tournier et al., 2019), Freesurfer (Fischl, 2012), DSI Studio (Yeh et al., 2013), Dipy (Garyfallidis et al., 2014), DiffusionKit (Xie et al., 2016), and SlicerDMRI (Norton et al., 2017; Zhang et al., 2020b).

**Table 1:** Summary of the main software used for the study of dMRIs.

**Software**	**Lenguaje**	**OS**	**Distribution license**	**dMRI format**	**Tractography format**
BrainSUITE	C++, Matlab	Multi.	Open-source	DICOM, NIfTI, Analyze	TRK
Camino	Java	Linux MacOs	Open-source	DICOM, NIfTI	VTK
Diffusion toolkit	C++	Multi.	Open-source	DICOM, NIfTI, Analyze	TRK
ExploreDTI	Matlab	Multi.	Non-commercial package	DICOM, NIfTI Analyze, Matlab formats	MAT
FSL	C++/Unix	Linux MacOs Windows	Non-commercial package	NIfTI	NIfTI
MRtrix	C++, OpenGL	Linux MacOs	open-source	DICOM, Analize NIfTI, MGH MRtrix formats	TCK
FreeSurfer	C/C++, Python, Matlab	Linux	Open-source	DICOM, Analyze NIfTI, MINC	-
DSI Studio	C++	Multi.	Open-source	DICOM, NIfTI	TRK
Dipy	Pyhton, Cython	Multi.	Open-source	Analyze, NIfTI, DICOM	TCK, TRK
DiffusionKit	C/C++	Windows Linux	Freely available	DICOM, NIfTI	TRK
SlicerDMRI	C++, Python	Multi.	Open-source	DICOM, Analyze, NIfTI, nrrd/nhdr	VTK

The order of the columns is as follows: Software, Lenguaje, OS, Distribution License, dMRI Format, and Tractography Format. OpenGL, Open graphics library; Multi., Multiplatform; NIfTI, Neuroimaging informatics technology initiative; DICOM, Digital imaging and communications in Medicine; MGH, FreeSurfer format; MINC, FreeSurfer format; VTK, Visualization ToolKit; TRK, Track File; Analyze, Image data format; TCK, Tracks file format; and MAT, Matlab file.

**Table 2:** Summary of the main software used for the study of dMRIs.

**Software**	**DW Model Reconst**.	**Fiber Track**.	**Fiber Clustering**	**Bundle Segment**.	**Visual-ization**	**Fiber Measur**.
BrainSUITE	DTI	Det.	-	-	Slice/Volume, DW model, tractography	-
Camino	DTI/ multifiber HARDI, QBall, PASMRI	Det. Prob.	-	-	Slice/Volume, DW model, tractography	-
Diffusion toolkit	DTI, DSI, QBI	Det.	-	-	Uses TrackVis	-
ExploreDTI	DTI, QBI, CSD	Det. Prob.	-	Using ROIs	Slice/Volume, DW model, tractography	Mean length
FSL	DTI	Prob.	-	-	Slice/Volume, Meshes	-
MRtrix	DTI, Single-tissue CSD, Multi-tissue CSD	Det. Prob.	-	-	Slice/Volume, DW model, tractography	-
FreeSurfer	TRACULA	Prob.	Anato-micCuts	TRACULA	Slice/Volume, Meshes	-
DSI Studio	DTI, DSI, QBI	Det.	-	Using ROIs	Slice/Volume, DW model, Tractography, Meshes	Count, mean length
Dipy	DTI, DSI, QBI, CSD	Det. Prob.	Quick-Bundles	Reco-
Bundles	Slice/Volume, DW model, Tractography, Meshes	Count, mean length
DiffusionKit	DTI, CSD, dec. -based SPFI	Det.	-	-	Slice/Volume, DW model, Tractography	-
SlicerDMRI	DTI, Multi-tensor UKF	Det.	-	Using ROIs	Slice/Volume, DW model, Tractography	Points numbers, count, mean length

The order of the columns is as follows: Software, DW (diffusion-weighted) Model Reconstruction, Fiber Tracking, Fiber Clustering, Bundle Segmentation, Visualization, and Fiber Measures. DTI, Diffusion tensor imaging; HARDI, High angular resolution diffusion Imaging; DSI, Diffusion spectrum imaging; QBI, Q-Ball imaging; CSD, Constrained spherical deconvolution; SPFI, Spherical polar Fourier imaging; TRACULA, TRActs Constrained by UnderLying Anatomy; Det., Deterministic; Prob., Probabilistic; ROIs, Regions of interest; QuickBundles, (Garyfallidis et al., 2012); AnatomicCuts, (Siless et al., 2018) RecoBundles (Garyfallidis et al., 2018); DW, Diffusion weighted; and UKF, unscented Kalman filter (Malcolm et al., 2010).

As shown in Tables 1, 2, the tool packages have different functionalities. Some of them are more focused on dMRI pre-processing, model reconstruction, and tractography, and others include fiber tractography analysis methods. It is common for users to employ more than one software to implement their processing pipeline, where special attention should be paid to file formats, the reference coordinate system (Tournier et al., 2019), and the common 3D space, when required. Of course, no tool contains all the existing algorithms, although there are some fairly comprehensive ones.

There are fewer software packages dedicated to analyzing tractography data, such as fiber clustering and segmentation, as well as filtering fiber clusters. Hence, we present a toolkit for the analysis of brain tractography data. The package combines several tools for tractography analysis that are available in the literature, developed by our group. These include the optimized fiber bundle segmentation algorithm using a brain fiber atlas (Guevara et al., 2012; Labra et al., 2017; Vázquez et al., 2019), the hierarchical fiber clustering (Román et al., 2017, 2022) and Fast Fiber Clustering (FFClust) based on K-Means (Vázquez et al., 2020). These tools are difficult to apply for external users due to the lack of unified code, the multiplicity of programming languages, the plurality of library dependencies, and the lack of example code/data and documentation. To overcome these issues, we developed an open-source library, called Phybers, that integrates all these algorithms, along with other fiber cluster analysis and visualization tools.

The algorithms included in the library were implemented in C/C++ and Python 3.9. It was structured into four modules: Segmentation, Clustering, Utils, and Visualization. The library was implemented in Python to efficiently provide easy manipulation of data and input parameters, to users without computer science background. Also, Python allows better interoperability with software such as the Dipy package (Garyfallidis et al., 2014). The library includes several internal functions written in C/C++ to reduce the execution time of computationally intensive calculations, such as Euclidean distances between pairs of fibers, which are accessible through Cython. Phybers is freely available and provides the documentation and test data for its execution.

## 2 Materials and methods

Phybers contains four modules that include algorithms for different pre-processing stages. The suite of Utils contains tools for pre-processing the tractography data prior to fiber segmentation or clustering, such as the transformation of fibers (*bundles* format) to another space using a deformation field (NIfTI format). The analysis modules include a fiber bundle segmentation algorithm based on a brain fiber bundle atlas (Guevara et al., 2012; Labra et al., 2017; Vázquez et al., 2019), and two clustering algorithms, Fast Fiber Clustering (FFClust) (Vázquez et al., 2020) and Hierarchical Clustering (HClust) (Román et al., 2017, 2022). Also, a set of post-processing tools is provided for the analysis of the results of the fiber bundle segmentation and fiber clustering algorithms (*bundles* format). The Visualization module supports different types of data such as volume (NIfTI), mesh (*mesh* and GIfTI formats), and fibers (TRK, TCK, and *bundles*). In addition, it integrates an interactive graphical user interface (GUI) that allows the user to manipulate 3D objects in real-time. For example, manual segmentation of brain fibers can be performed by positioning two or more 3D regions.

**Figure 1:**
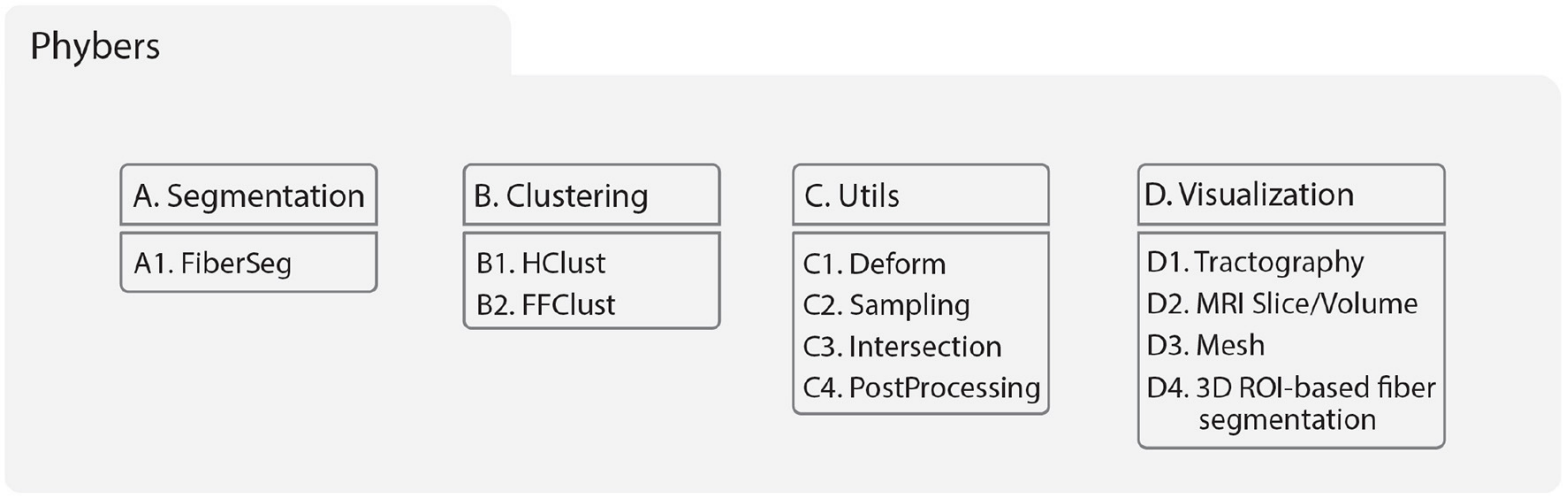
The hierarchical library structure was separated into four modules: **(A)** Segmentation, **(B)** Clustering, **(C)** Utils, and **(D)** Visualization. **(A)** Segmentation contains the fiber bundle segmentation algorithm using a white matter fiber bundle atlas (A1. FiberSeg); refer to Section 2.2.1. **(B)** Clustering includes an average-link hierarchical clustering based on the Euclidean distance among fiber pairs (B1. HClust) and Fast Fiber Clustering (B2. FFClust), refer to Section 2.2.2. **(C)** Utils module includes several tools for analyzing brain fibers, such as C1. Deforms: transformation of fibers to another space using a deformation field, C2. Sampling: sampling of fibers with *n* equidistant points, C3. Intersection: intersection between two sets of fibers, and C4. PostProcessing: Calculates the size and length of a set of fibers and the maximum Euclidean distance between fibers of a set; refer to Section 2.2.3. **(D)** Visualization module is a tool for rendering multiple data types such as brain fibers, MRI slices/volumes, meshes, and fibers selection manually by ROI; refer to Section 2.2.4.

Phybers was developed in Python to distribute and update in a PyPI repository. We implemented the algorithms in C/C++ and Python 3.9, which used Python dependencies such as numpy (Harris et al., 2020), nibabel, pandas, and subprocess. However, all dependencies are automatically installed with the package. Library installation can be performed using the command *$ pip install phybers*, and the software distribution includes sample data with code examples for all supported functionalities. Phybers is compatible with Python versions higher than Python 3.9 and supports Python platforms such as Jupyter Notebook and Spyder, providing greater flexibility to cater to the specific needs of each user. Additionally, it functions seamlessly on both Ubuntu and Windows systems and can also be utilized on macOS via a virtual machine. Finally, the library documentation was generated with Sphinx. Phybers library was structured into four modules (Figure 1) defined as Segmentation, Clustering, Utils, and Visualization. The following sections describe the library modules.

The data used to showcase the examples correspond to a random subject from the HCP database (Glasser et al., 2013). Specifically, preprocessed diffusion images (“data.nii.gz”) and deformations to the MNI (Montreal Neurological Institute) space (“acpc_dc2standard.nii”) were utilized. Deterministic tractography calculations were performed using DSI Studio software (Yeh et al., 2013) with GQI model reconstruction. Two datasets of brain tractography were calculated. The first dataset was generated using the following fiber tracking parameters: angular threshold = 60°, step size = 0.5 *mm*, smoothing = 0.5, minimum length = 30 mm, maximum length = 300 *mm*, and a tract count of 1.5 million fibers. The second brain tractography dataset was obtained by placing ROIs, in this case, using the postcentral region from the FreeSurfer Aseg Atlas (Fischl et al., 2002), with the same fiber tracking parameters, except for the minimum length = 90 *mm*, maximum length = 130 *mm*, and a tract count of 4,000 fibers.

### 2.1 Data structure and format

#### 2.1.1 Tractography datasets

Brain tractography datasets are sets of 3D polylines, also called streamlines or fibers. A tractography file contains arrays with the coordinates of the fiber 3D points and may include other metadata, such as an affine transformation. These files can be read in Python through different libraries, depending on the file format, as a list of numpy arrays. The most commonly used formats are TRK, TCK and *bundles*. In the proposed library, we use the *bundles* format. Several functions are provided to read and write fibers in this format. The advantage of *bundles* format is the support of the labeling of bundles, i.e., a single file can contain several bundles or clusters, reducing the computational cost for reading/writing and visualization. This format uses two files: the text metadata file *.bundles* that contains the bundle labels, and the binary *.bundlesdata* file, which contains the 3D coordinates of the fiber points. Using available readers and writers of other formats, it is possible to convert fiber tractography datasets from different formats. Therefore, we included a source code in the Phybers documentation that enables the conversion of brain tractography dataset from the TRK format (used by TrackVis and DSI Studio, among others) to the *bundles* format. Additionally, this code facilitates the conversion of TCK format (used by MRtrix and others) to the *bundles* format. We also shared source code for converting from *bundles* format to TRK and TCK formats. More information and access to these codes can be found in the Phybers documentation.

#### 2.1.2 MRI

MRI volumes are 3D arrays, which can be read in Python as a 3-dimensional numpy array. The most commonly used formats are NIfTI, Analyze, and DICOM. In the proposed library, the NIfTI format is used to read MRI images.

#### 2.1.3 Mesh

Meshes are geometric surface objects, that can be read in Python as an array of vertices and an array of triangles (vertex indices). The most commonly used formats are GIfTI and *mesh*. The proposed library uses the GIfTI and *mesh* formats for the meshes.

### 2.2 Library hierarchy

#### 2.2.1 Segmentation module

**Figure 2:**
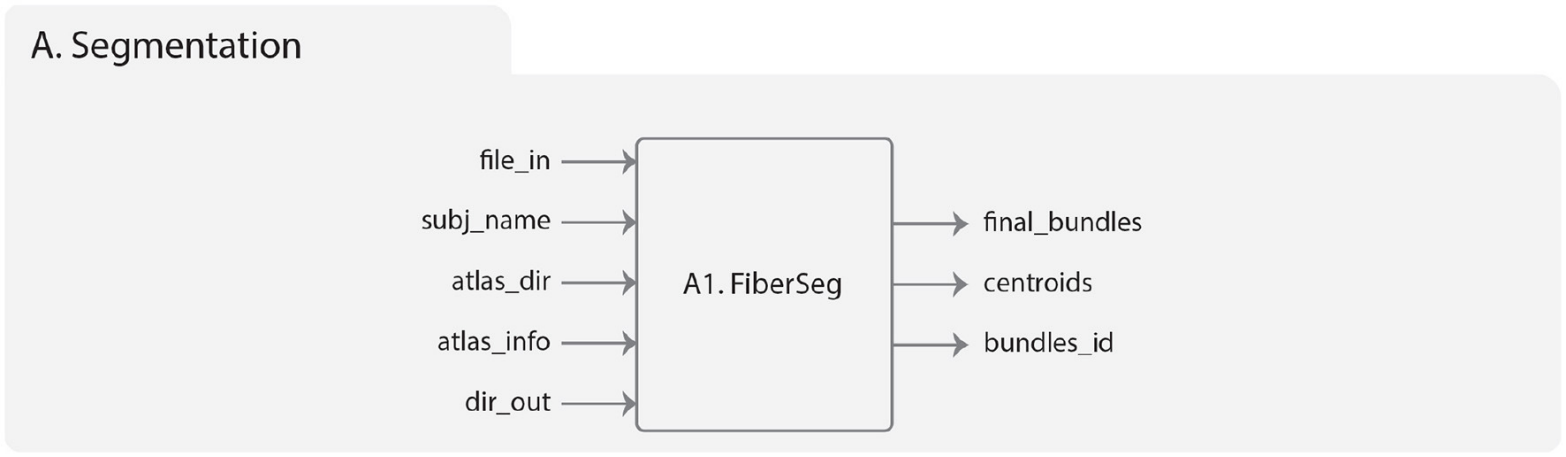
Diagram representing the Segmentation module. On the **left** is represented the input data, which includes the brain fibers to be segmented (file_in), subject name (subj_name), the atlas of bundles (atlas_dir), the atlas threshold (atlas_info), and the result directory (dir_out). On the **right** are the output folders, which include the segmented brain fibers, the centroids of the segmented fibers, and the index of each fiber grouped by fasciculus.

This module includes a white matter fiber bundle segmentation algorithm (Guevara et al., 2012; Labra et al., 2017; Vázquez et al., 2019) based on a multi-subject atlas (Figure 2). The method uses as a measure of similarity between pairs of fibers the maximum Euclidean distance between corresponding points (*d*_*ME*_), defined as:


(1) $$\begin{array}{l} d_{ME}\left(A,B\right)=min\left(max_{i}\left(\left|a_{i}-b_{i}\right|\right),max_{i}\left(\left|a_{i}-b_{N_{p}-i}\right|\right)\right) \end{array}$$

Where *a*_*i*_ and *b*_*i*_ represent the 3D coordinates of the points in fibers A and B, respectively, both having an equal number of points (*N*_*p*_), listed in direct order. Here the points of fiber A are sequentially traversed as *a*_*i*_ = [*a*_1_, *a*_2_, …, *a*_*N*_*p*__], and those of B are similarly defined as *b*_*i*_ = [*b*_1_, *b*_2_, …, *b*_*N*_*p*__]. Therefore, the reverse order of fiber B is expressed as *b*_*N*_*p*−*i*__ = [*b*_*N*_*p*__, *b*_*N*_*p*−1__, …, *b*_1_].

The original version was written in Python and presented in Guevara et al. (2012). It aims at classifying the subject fibers according to a multi-subject bundle atlas. The bundle atlas consists of a set of representative bundles and additional information. The fibers of the atlas bundles are called centroids. We include one atlas of deep white matter (DWM) bundles (Guevara et al., 2012) and two atlases of superficial white matter (SWM) bundles (Román et al., 2017, 2022). These atlases are located in the MNI space (aligned with “ICBM 2009a Nonlinear Symmetric” template) and are available for download from the Phybers github repository. We have also tested the algorithm using the DWM and SWM bundle atlas of Zhang et al. (2018).

The fibers of each subject are classified using a maximum *d*_*ME*_ distance threshold for each bundle between the subject's fibers and the atlas centroids. The fibers are labeled with the closest atlas bundle, given that the distance is smaller than the distance threshold (in *mm*). The algorithm was progressively improved first by Labra et al. (2017) that developed a fast fiber discarding algorithm in C language. Then, it was optimized by Vázquez et al. (2019) using a C++ parallel implementation.

The white matter fiber bundle segmentation algorithm based on a multi-subject atlas included in Phybers is called FiberSeg (Figure 2A1) and is based on the implementation by Vázquez et al. (2019). Among the noteworthy enhancements is that this algorithm is compatible with both Ubuntu and Windows, unlike the previous version that only supported Ubuntu. New functionalities have been added, allowing for the extraction of bundle centroids and indices of original fibers per bundle. This addition proves beneficial for fiber bundle segmentation on the subject space and facilitates the calculation of diffusion tensor-derived measures (Basser et al., 1994) (FA, MD, AD, RD). In this version, the algorithm accepts fibers with a variable number of points, unlike the previous version that fixed the point count at 21 for input data. Furthermore, the data structure has been improved, enabling the loading of larger input tractography datasets and brain fiber atlases. Overall, enhanced data structures have been defined to optimize memory usage.

The implementation of FiberSeg in the Segmentation module of Phybers has the following inputs (Figure 2):

1. ***file_in***: the whole-brain tractography dataset file of a subject. The fibers must be in the same reference system as the used bundle atlas and be in *bundles* format.
2. ***subj_name***: subject name, used to label the results.
3. ***atlas_dir***: the bundle atlas folder, with bundles in separate files, sampled at 21 equidistant points.
4. ***atlas_info***: a text file associated to the used atlas, that stores information needed to apply the segmentation algorithm, i.e., a list of the atlas fascicles, containing the name, the segmentation threshold (in *mm*) and the size of each fascicle. Note that the segmentation threshold can be adjusted depending on the database to be used.
5. ***dir_out***: the directory name to store all the results generated by the algorithm.

FiberSeg outputs are:

1. ***final_bundles***: the directory with the segmented fibers, i.e., the atlas fascicles extracted from the subject's tractography dataset, which are labeled and saved in separate files in *bundles* format.
2. ***centroids***: a directory that contains the centroid of each segmented fascicle, saved in a single file in *bundles* format.
3. ***bundles_id***: a text file containing, for each segmented bundle, the indexes of the fibers in the subject's tractography dataset file.

**Figure 3:**
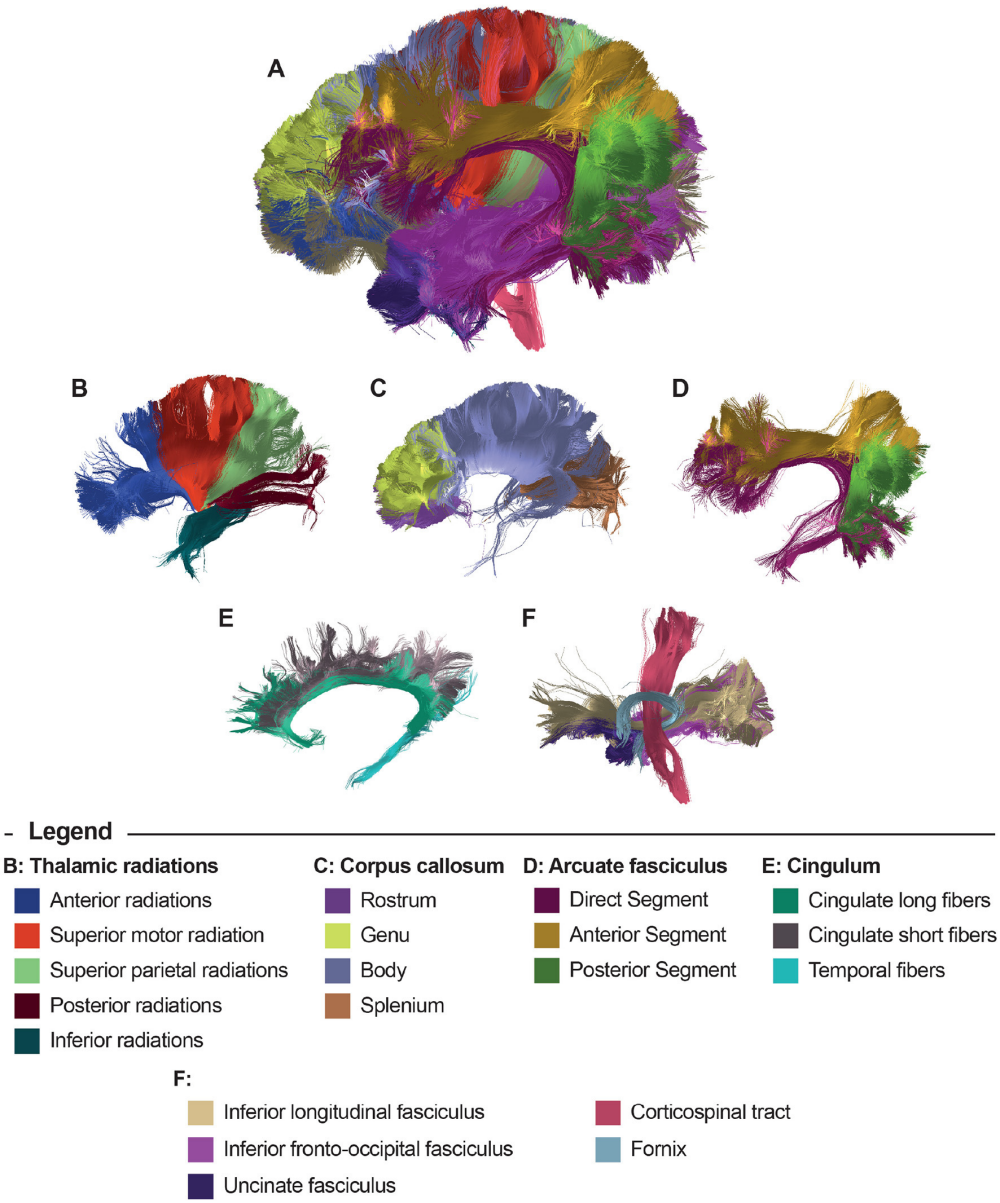
Bundle segmentation results for a subject using the DWM bundle atlas (Guevara et al., 2012). **(A)** Sagittal view of the whole-brain segmentation. **(B)** Thalamic radiations, **(C)** Corpus callosum segments, **(D)** Arcuate fasciculus, **(E)** Cingulum fibers, and **(F)** Inferior longitudinal fasciculus, Inferior fronto-occipital fasciculus, Uncinate fasciculus, Corticospinal tract, and Fornix.

**Figure 4:**
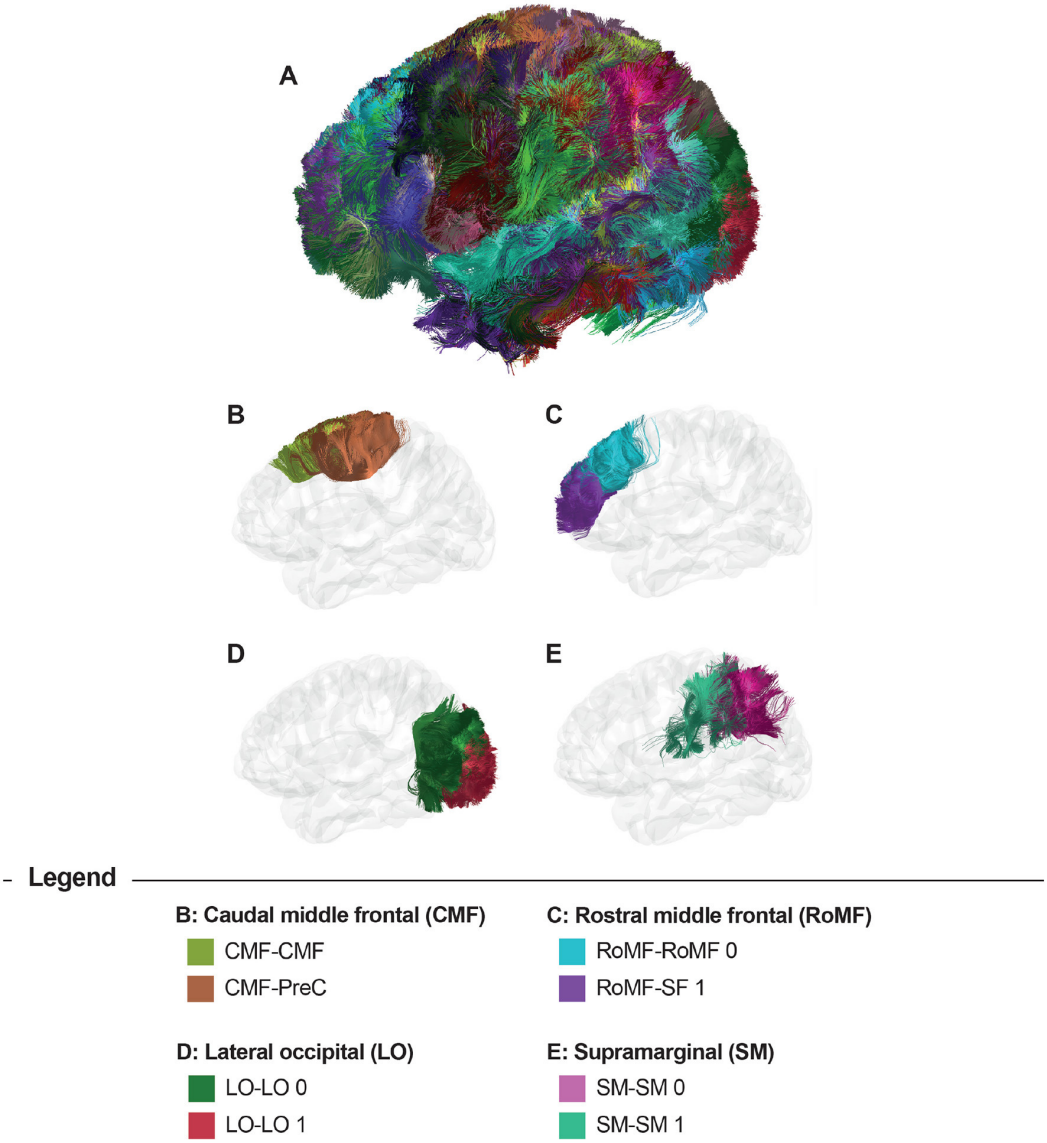
Bundle segmentation results for a subject using the SWM bundle atlas (Román et al., 2017). **(A)** Sagittal view of the whole-brain segmentation. **(B)** Caudal middle frontal (CMF) and CMF - Precentral (PreC) bundles (CMF-CMF, CMF-PreC), **(C)** Rostral middle frontal (RoMF) and RoMF - Superior frontal (SF) bundles (RoMF-RoMF 0, RoMF-SF 1), **(D)** Lateral occipital (LO) bundles (LO-LO 0, LO-LO 1), and **(E)** Supramarginal (SM) bundles (SM-SM 0, SM-SM 1).

Figure 3 displays the results of the bundle segmentation using the DWM bundle atlas Guevara et al. (2012) for a subject from the HCP database. The segmented bundles shown are Thalamic radiations (B), Corpus callosum segments (C), Arcuate fasciculus (D), Cingulum fibers (E), Inferior longitudinal fasciculus, Inferior fronto-occipital fasciculus, Uncinate fasciculus, Corticospinal tract, and Fornix (F). Figure 4 shows the segmentation results using a SWM bundle atlas (Román et al., 2017). This atlas comprises 93 fascicles, labeled based on anatomical ROIs extracted from the Desikan-Killiany atlas (Desikan et al., 2006). Four groups of short association fiber bundles are presented in more detail: Caudal middle frontal (B), Rostral middle frontal (C), Lateral occipital (D), and Supramarginal (E) bundles.

#### 2.2.2 Clustering module

##### 2.2.2.1 HClust sub-module

HClust (Hierarchical Clustering) (Román et al., 2017, 2022), is an average-link hierarchical agglomerative clustering algorithm that creates bundles based on a pairwise fiber distance measure. It is implemented in Python and C++. The algorithm calculates a distance matrix between all fiber pairs for a bundles dataset (*d*_*ij*_), by using the maximum Euclidean distance between fiber points (Equation 1). Then, it computes an affinity graph on the *d*_*ij*_ matrix for fiber pairs that have a Euclidean distance below a maximum distance threshold (*fiber_thr*) in *mm*. The affinity is given by Equation (2) (Donnell and Westin, 2007),


(2) $$\begin{array}{l} a_{ij}=e^{\frac{-d_{ij}}{\sigma^{2}}} \end{array}$$

Where *d*_*ij*_ is the distance between the elements *i* and *j*, and σ is a parameter that defines the similarity scale in *mm*.

From the affinity graph, the hierarchical tree is generated using an agglomerative average-link hierarchical clustering algorithm. The tree is adaptively partitioned using an intra-cluster distance threshold (*partition_thr*) in *mm*.

The version of the Hierarchical Clustering developed in Phybers is based on the work of Román et al. (2017), that was improved in Román et al. (2022), utilizing a C++ implementation of the agglomerative clustering algorithm proposed in the Python Sklearn library. Our implementation (HClust) allows for calculating centroids of obtained clusters and records indices of original fibers belonging to each detected cluster. Additionally, it is compatible with both Windows and Ubuntu, overcoming a limitation present in the previous version that was exclusively operational on Ubuntu.

**Figure 5:**
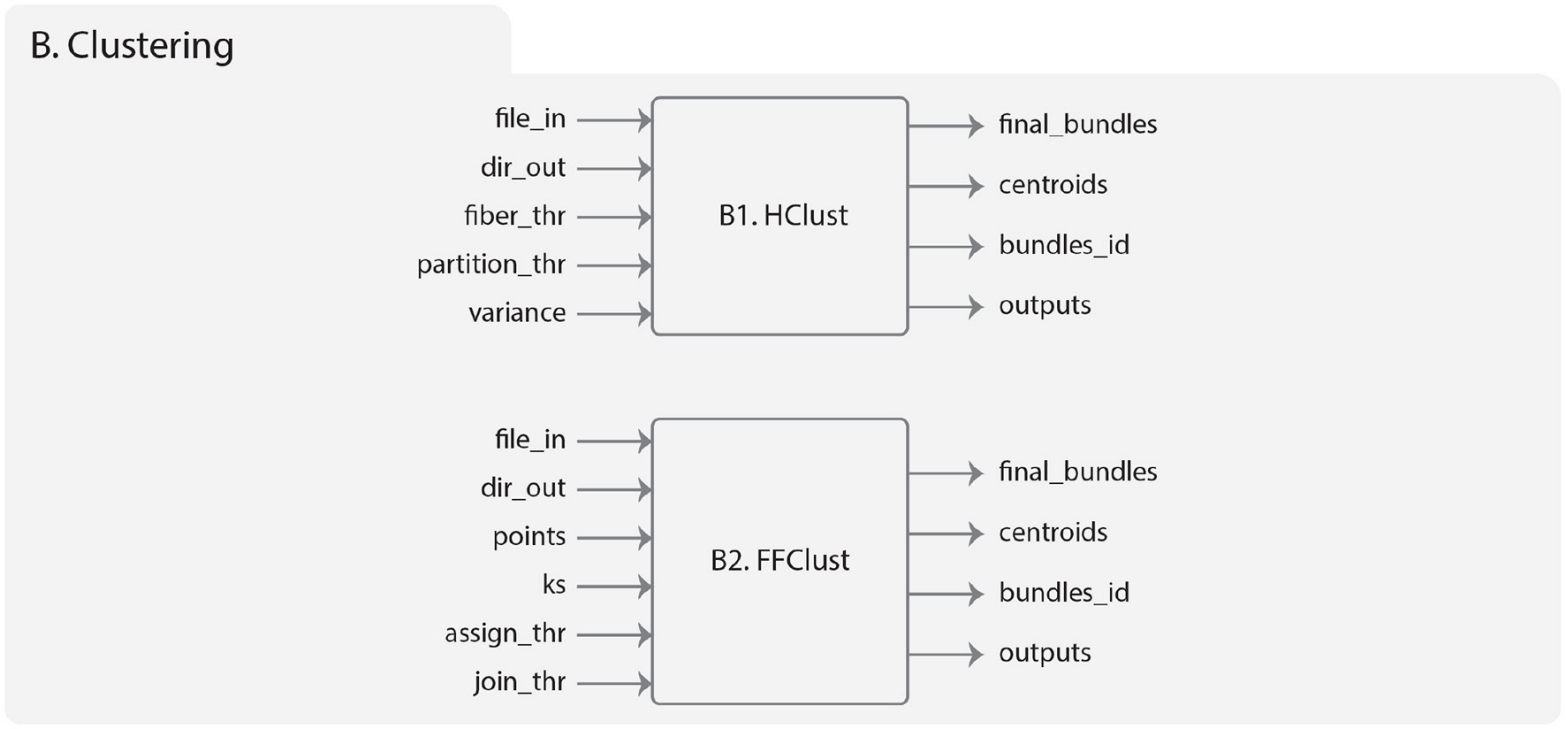
Diagram representing the Clustering module. **(B1)** Describes the HClust algorithm. On the left is represented the input data, which includes the brain fibers to be clustered (file_in), the result directory for saving the outputs (dir_out), the maximum distance threshold (fiber_thr, in *mm*), the threshold for dendrogram partitioning (partition_thr, in *mm*), and variance is measured on a similarity scale in *mm*. On the right are the output folders, which include the obtained fiber clusters, the centroids of the clusters, the index of the fibers of each cluster, and the temporal directory with intermediate results. **(B2)** Describes the FFClust algorithm. On the left is represented the input data, including the brain fibers to be clustered (file_in), the result directory for saving the outputs (dir_out), the numbers of fiber points to be used in the clustering (points), the number of clusters used by Minibatch K-Means for each chosen fiber point (ks), the threshold distance for reassigning points to a cluster (assign_thr, in *mm*), and the threshold distance for merging clusters (join_thr, in *mm*). On the right are the output folders, which include the obtained fiber clusters, the centroids of the clusters, the index of the fibers of each cluster, and the temporal directory with intermediate results.

The inputs of HClust are the following (Figure 5B1):

1. ***file_in***: the input tractography data file.
2. ***dir_out***: the directory to store all the results generated by the algorithm.
3. ***fiber_thr***: a maximum distance threshold (in *mm*), default 30*mm*.
4. ***partition_thr***: an adaptive partition threshold (in *mm*), default 40 *mm*.
5. ***variance***: a similarity scale (in *mm*), default 60 *mm*.

HClust outputs are:

1. ***final_bundles***: the directory that stores all the generated fiber clusters that are labeled with the cluster number and saved in separate files in *bundles* format.
2. ***centroids***: a directory that contains the centroids for each created cluster, saved in a single file in *bundles* format.
3. ***bundles_id***: a text file storing for each cluster the indexes of the fibers in the subject's tractography dataset file.
4. ***outputs***: a temporal directory with intermediate results.

**Figure 6:**
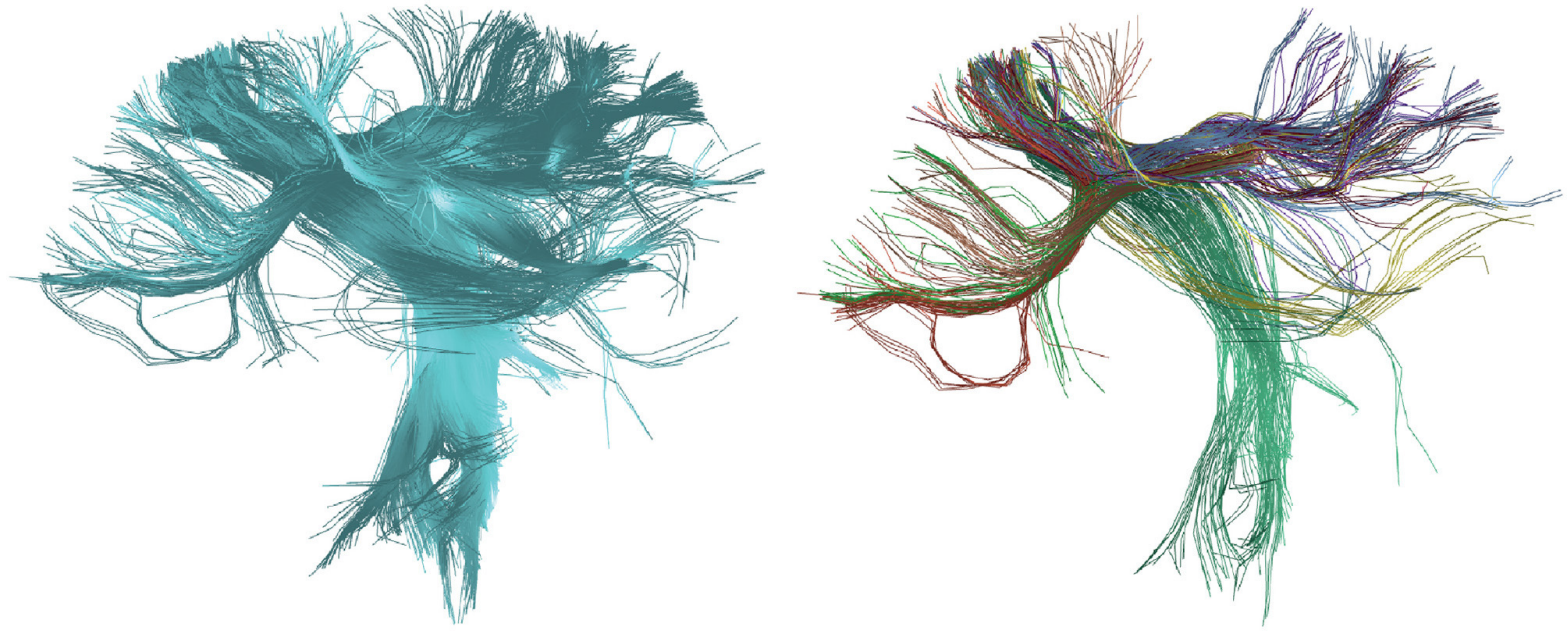
Results of the HClust algorithm for the calculated tractography dataset of the postcentral region. **(Left)** In blue, the reconstructed fibers for the postcentral region are shown before applying clustering. **(Right)** Displays eight clusters manually chosen from the total detected fiber clusters with random colors.

Figure 6 illustrates the results of applying the HClust algorithm to a tractography dataset of 4,000 fibers. On the left, the tractography with 4,000 fibers is presented in blue before clustering, and on the right, eight detected fiber clusters are shown, manually chosen and using a palette of random colors. In this case, the size of the brain tractography dataset has been reduced due to the high computational cost associated with the HClust algorithm. This challenge is attributed to the distance matrix calculated at the start of the algorithm, serving as its primary limitation. We recommend using HClust on tractography datasets of a maximum of 40,000 fibers. If applying it to the entire brain with a larger dataset is desired, one can consider the strategy of first utilizing the intra-subject clustering of FFClust and then applying HClust to the centroids of FFClust (Román et al., 2022).

##### 2.2.2.2 FFClust sub-module

FFClust (Fast Fiber Clustering) (Vázquez et al., 2020) is an intra-subject clustering algorithm that aims to identify compact and homogeneous fiber clusters on a large tractography dataset. The algorithm consists of four stages. First, it applies the Minibatch K-Means clustering on five specific fiber points (Stage 1), and merges fibers sharing the same point clusters (map clustering) (Stage 2). Next, it reassigns small clusters to bigger ones (Stage 3), considering the distance of fibers in direct and reverse order. Finally, the algorithm groups clusters sharing the central point and merges close clusters represented by their centroids (Stage 4). The distance among fibers is defined as the maximum Euclidean distance between the corresponding fiber points. The algorithm supports sequential and parallel execution using OpenMP.

The implementation of FFClust in Phybers is based on the work of Vázquez et al. (2020). This version brings improvements, such as handling variable sizes of brain fibers. Previously, the number of points was fixed at 21 for input data. Additionally, FFClust is now compatible with both Windows and Ubuntu platforms, overcoming the previous limitation that restricted its exclusive use on Ubuntu platforms. Figure 5B2 shows the hierarchy of the module. The inputs are:

1. ***file_in***: the input tractography dataset file.
2. ***dir_out***: the directory to store all the results generated by the algorithm.
3. ***points***: the index of the points to be used in the point clustering (Stage 1), default: 0, 3, 10, 17, 20.
4. ***ks***: the number of clusters to be computed for each point using K-Means (Stage 1), default: 300, 200, 200, 200, 300.
5. ***assing_thr***: a maximum distance threshold for the cluster reassignment in *mm* (Stage 3), default: 6.0 mm.
6. ***join_tht***: a maximum distance threshold for the cluster merge in *mm* (Stage 4), default: 6.0 mm.

The structure of FFClust outputs is similar to HClust module.

**Figure 7:**
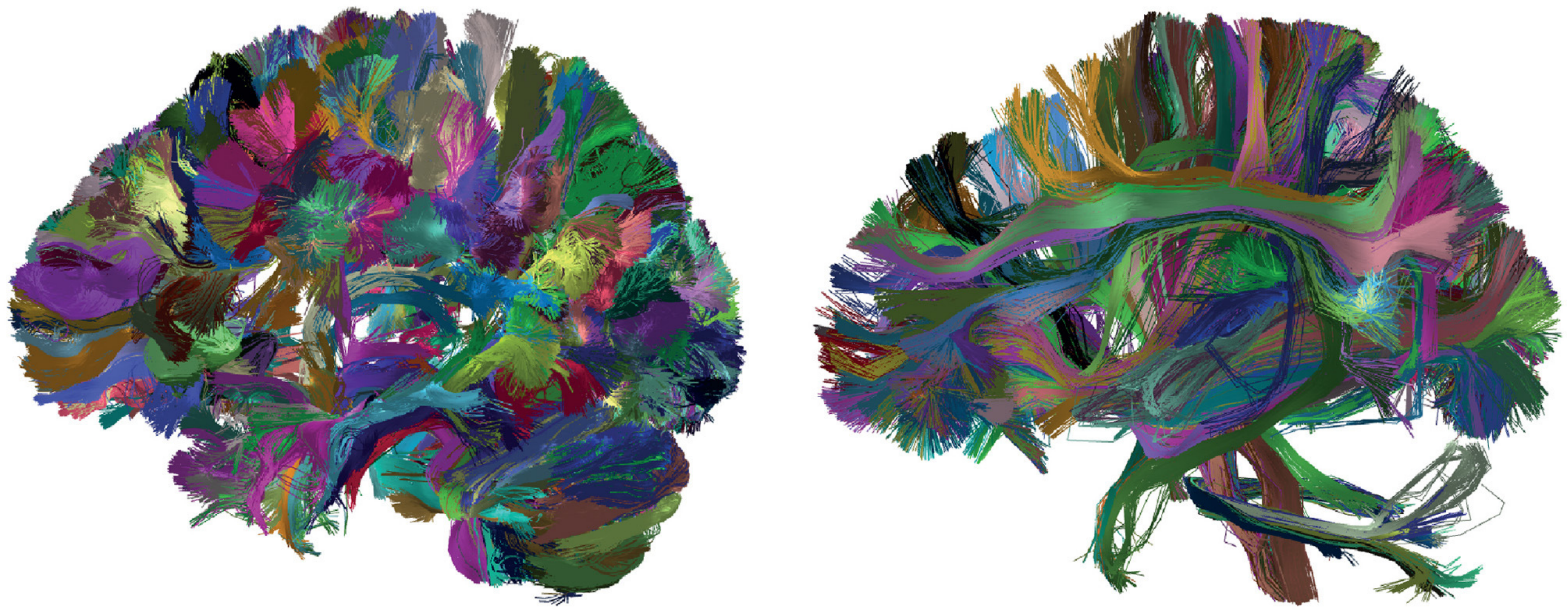
Results obtained with the FFClust algorithm. Fiber colors are randomly distributed, and all fibers within a cluster are assigned the same color. **(Left)** Clusters with a size greater than 150 and a length ranging from 50 to 60 *mm*. **(Right)** Clusters with a size greater than 100 and a length exceeding 150 *mm*.

Figure 7 shows the results of applying FFClust to the whole-brain tractography dataset with 1.5 million streamlines. The detected clusters were filtered using the PostProcessing sub-module of the Utils module (Section 2.2.3) to simplify result visualization. Clusters with a size greater than 150 and a length between 50 and 60 *mm* are shown on the left side of the figure, while clusters with a size greater than 100 and a length greater than 150 *mm* are shown on the right side of the figure.

#### 2.2.3 Utils module

The Utils module is a set of tools used for tractography dataset pre-processing and the analysis of brain fiber clustering and segmentation results. The module includes tools for reading and writing brain fiber files in *bundles* format, transform the fibers to a reference coordinate system based on a deformation field, sampling of fibers at a defined number of equidistant points, calculation of intersection between sets of brain fibers, and tools for extracting measures and filtering fiber clusters or segmented bundles. We considered the extraction of measures such as size, mean length (in *mm*), and the distance between fibers of each cluster (or fascicle), in *mm*. The set of tools implemented in Utils is being introduced for the first time in Phybers, and the source code is mostly developed in C/C++.

##### 2.2.3.1 Deform sub-module

**Figure 8:**
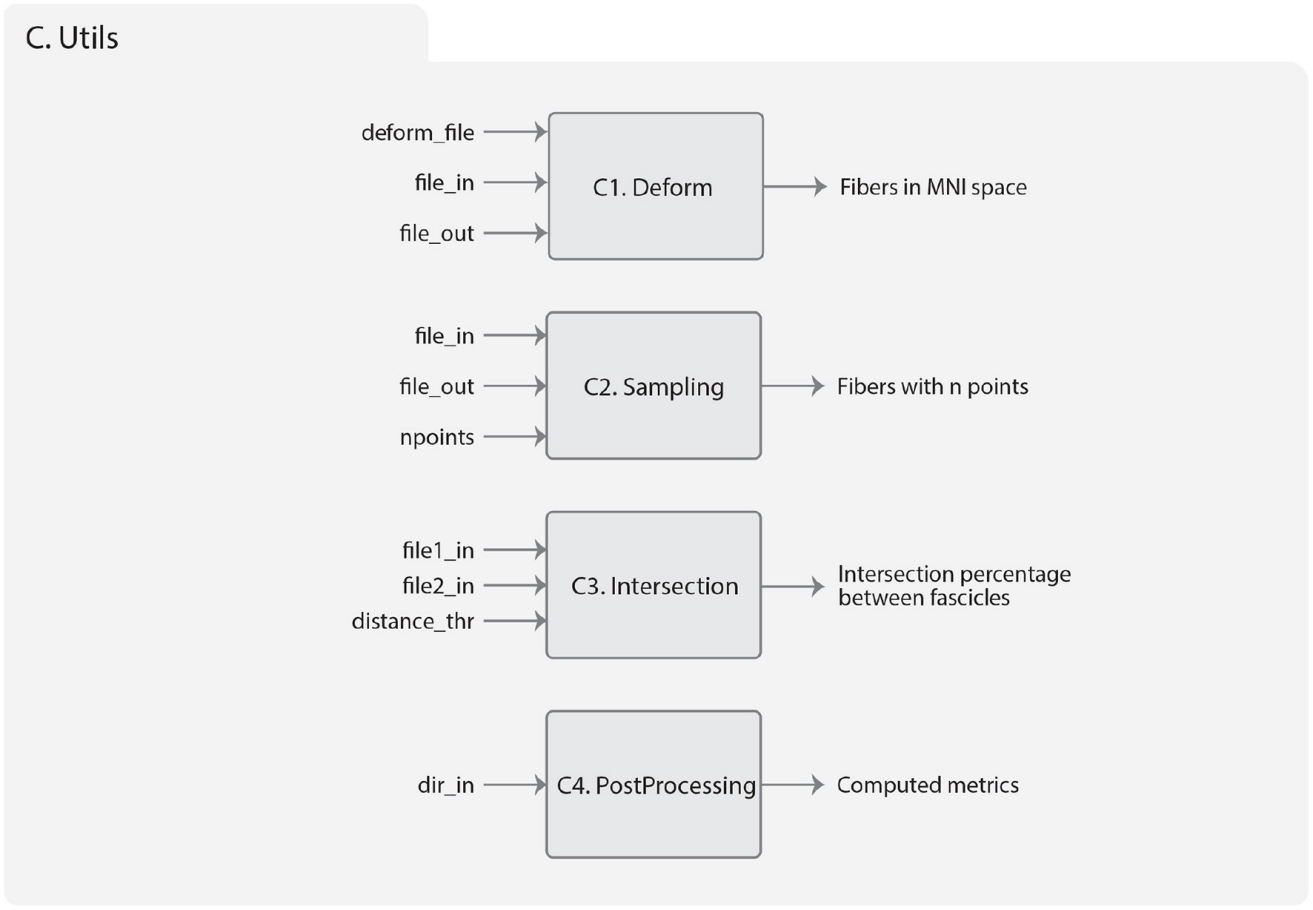
Diagram representing the Utils module. **(C1)** Deform sub-module, which has the inputs: deform_file (image in NIfTI format containing the deformations), file_in (path of the input tractography dataset), and file_out (path to the transformed tractography dataset). The output consists of a tractography dataset transformed into the MNI space. **(C2)** Sampling sub-module, which has the file_in (path input tractography dataset), file_out (path to save the sub-sampled fibers), and npoint (number of sampling points). The output is a tractography dataset sampled at n equidistant points. **(C3)** Intersection sub-module, which has as input the file1_in (path of the first fiber bundle), file2_in (path of the second fiber bundle), and distance_thr (in *mm*) used to consider similar two fibers. The output is a tuple object of Python with the percentage of intersections between the bundles. **(C4)** PostProcessing sub-module that has as input the dir_in where the segmentation or clustering result is located. The algorithm output includes information about the fiber size, bundle length (in *mm*), and intra-bundle fiber distance (in *mm*), all of which can be accessed through a DataFrame (Pandas object of Python).

The deformation sub-module (Figure 8C1) transforms a tractography dataset file to another space using a non-linear deformation file. The maps must be stored in NIfTI format, where the voxels contain the transformation to be applied to each voxel 3D space location. The Deform sub-module applies the deformation to the 3D coordinates of the fiber points. Deform needs as input data the deformation map, the file path of the fibers to be transformed, and the path of the output file, containing the tractography dataset file in the transformed space.

**Figure 9:**
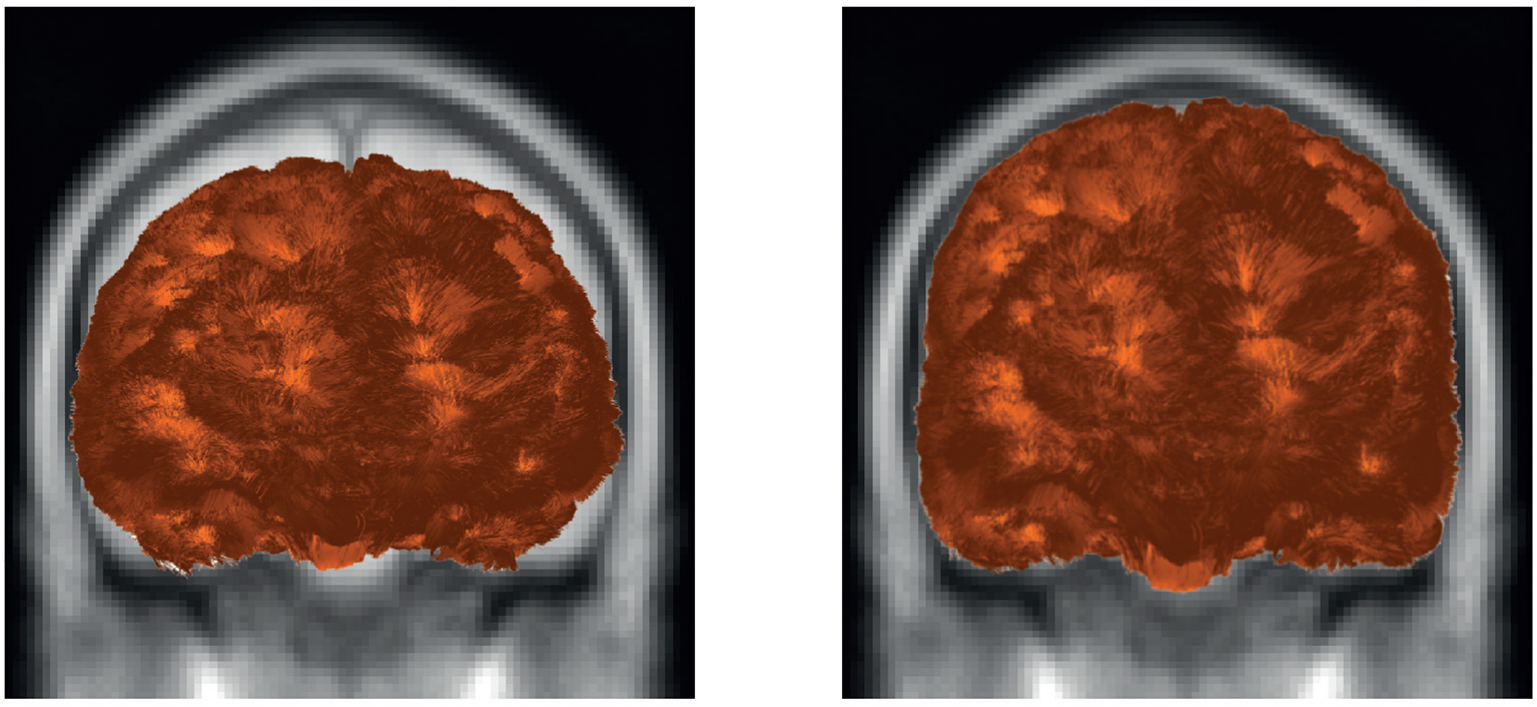
Example of fiber transformation using Deform sub-module. The tractography dataset before **(left)** and after **(right)** applying the transformation to the MNI space of a subject.

Figure 9 shows the result of applying the deformation function on a tractography dataset, using an anatomical image as visualization reference. The left side of the figure shows the tractography dataset before applying the transformation, and the right side shows the tractography dataset transformed to the MNI space. On the left side, there is a disalignment between the image and the tractography dataset, which is corrected on the right side.

##### 2.2.3.2 Sampling sub-module

Tractography datasets are usually composed of a large number of 3D polylines with a variable number of points. The Sampling sub-module (Figure 8C2) performs a sampling of the fibers, recalculating their points using a defined number of equidistant points. The input data of the algorithm are the path of the tractography dataset file to be sampled, the output file with the fibers with *n* points, and the number of points (*npoints*). The Sampling sub-module is used in the pre-processing stage of the segmentation and clustering algorithms.

##### 2.2.3.3 Intersection sub-module

The Intersection sub-module (Figure 8C3) calculates a similarity measure between two sets of brain fibers, that could be generated with other algorithms, such as fiber clustering (fiber clusters) and bundle segmentation (segmented bundles). It uses a maximum distance threshold (in *mm*) to consider two fibers as similar. Both sets of fibers must be in the same space. First, an Euclidean distance matrix is calculated between the fibers of the two sets. The number of fibers from one set that have a similar fiber in the other set are counted, for both sets. The similarity measure yields a value between 0 and 100%. The input data of the intersection algorithm are the two sets of fibers and the maximum distance threshold, while the output is the similarity percentage.

##### 2.2.3.4 PostProcessing sub-module

The PostProcessing sub-module (Figure 8C4) contains a set of algorithms that can be applied to the results of clustering and segmentation algorithms. This algorithm constructs a Pandas library object (Dataframe), where each key corresponds to the name of the fiber set (cluster or segmented fascicle), followed by measures defined on the fiber set such as number of fibers (size), intra-fiber bundle distance (in *mm*) and mean length (in *mm*). It can be used to perform single or multiple feature filtering on the clustering or segmentation results. The input of the algorithm is the directory with the bundle sets to be analyzed, and the output is a Pandas Dataframe object with the calculated metrics (Figure 8C4).

#### 2.2.4 Visualization module

**Figure 10:**
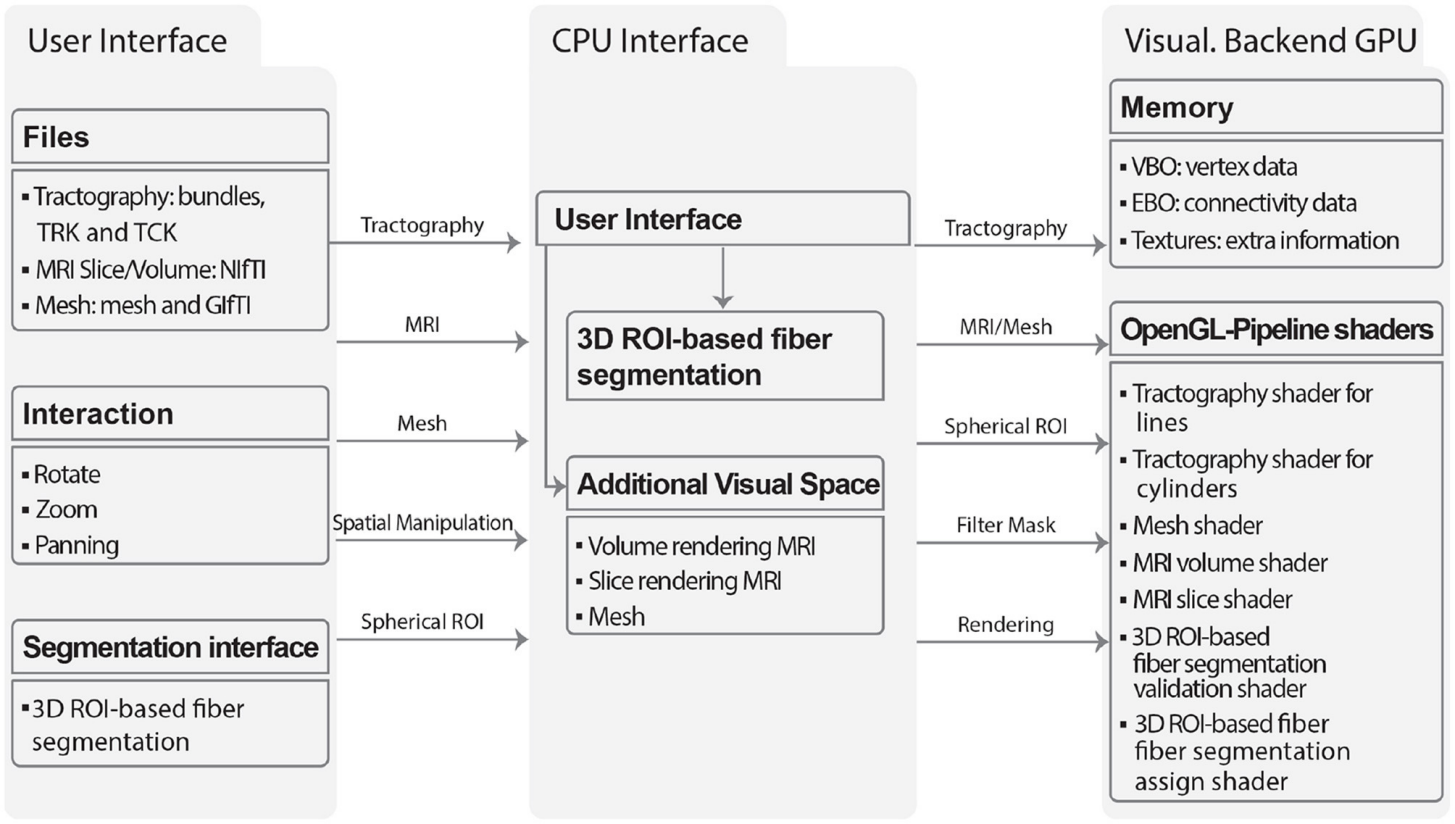
The flowchart illustrates the main components and interactions of the Visualization module: User Interface, CPU Interface, and Visualization Backend GPU. The graphical user interface defines the supported file formats for each object type (tractography datase, slice or volume images, and meshes), as well as the available interactions (including rotation, zoom, and panning) and segmentation (3D ROI-based fiber segmentation). The CPU interface facilitates the loading of objects and interactions into memory, which can be displayed through the Visualization Backend GPU using OpenGL pipeline shaders.

The Visualization module can render multiple types of 3D objects, including tractography dataset, meshes, and MRI scans as slices or volumes. The module was designed with a focus on scalability, utilizing dictionaries to store the objects to be displayed, thus enabling the rendering of multiple objects simultaneously. For each object, a set of functionalities is defined that can be accessed through a graphical user interface (GUI). The GUI enables visualization of multiple objects at once, performing camera operations such as zooming, rotating, and panning, modifying object material properties such as color and transparency, and applying linear transformations to brain tractography dataset. Figure 10 illustrates the flow diagram of the visualization module. The input data is read into RAM and processed by the CPU. This data is then loaded into the VRAM and rendered using shaders. MRI volumes are loaded as VBOs (Vertex Buffer Objects), EBOs (Element Buffer Objects), and textures, which are accessed during the rendering process. Tractography dataset files are loaded according to the bundle dataset format (*bundles*, TRK, TCK). ROI inputs are created through the user interface. MRI volume inputs consist of 3D images in the NIfTI format. Mesh inputs are loaded from GIfTI and *mesh* files. The GPU renders the objects using geometric primitives such as points, lines, and triangles, and it can also accept buffer objects as input. The EBO contains geometrical information, specifying which vertices form which primitives. The OpenGL pipeline and shaders are employed to offload computational tasks from the CPU to the GPU.

##### 2.2.4.1 Algorithms for visualization

The tractography dataset files can be rendered with lines or cylinders. In the case of lines, the software loads the streamlines, defining a fixed normal per vertex, which corresponds to the normalized direction for the particular segment of the streamline. Furthermore, a Phong lighting algorithm (Osorio et al., 2021) is implemented in a vertex shader to compute the color of the streamline. The MRI data is rendered using specific shaders for slice visualization and volume rendering. Meshes can be displayed using points, wireframes, or shaded triangles. The visualization algorithm, along with all its functionalities, such as the Interactive 3D ROI-based fiber segmentation, has been implemented for the first time for personal computers in Phybers.

##### 2.2.4.2 Interactive 3D ROI-based fiber segmentation

This function allows users to interactively extract fiber bundles using spherical ROIs. Internally, it creates a point-based data structure (Octree) for fast queries, based on storing points inside a bounding box with a capacity of N. When a node is filled, and a new point is added, the node subdivides its bounding box into eight new non-overlapping nodes, and the points are moved into the new nodes.

**Figure 11:**
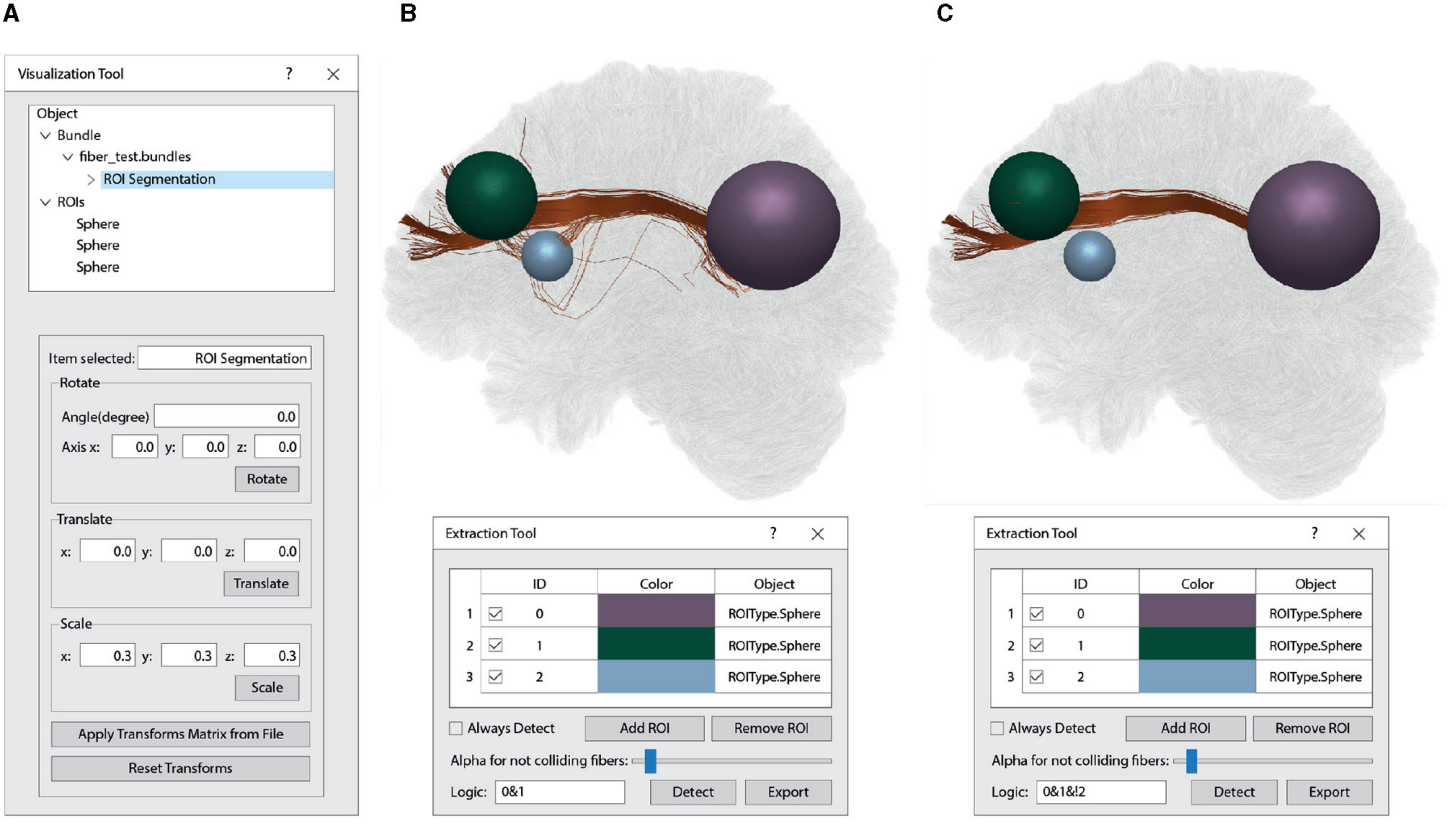
Interactive 3D ROI-based fiber segmentation using the Visualization Module. **(A)** Within the *Visualization Tool* window, the first quadrant is utilized to interact with objects loaded by the software. It shows a loaded tractograohy file (“fiber_test.bundles”) containing 1.5 million fibers. This fiber bundle dataset has undergone ROI segmentation, highlighted in blue. The ROIs object consists of three spheres organized in order of creation (0: purple, 1: green, and 2: blue). In the second quadrant of the Visualization Tool, various spatial manipulations, such as rotation, translation, and scaling, can be applied manually or through a text file (*Apply Transforms Matrix from File* and *Reset Transforms*). The manipulation options displayed depend on the selected object. **(B)** This section illustrates the segmentation of brain fibers connecting the purple and green spheres. Within the *Extraction Tool* window, you have access to various tools for interacting with the segmentation process, including *Always Detect* (real-time segmentation), *Add ROI, Remove ROI, Alpha for not colliding fibers* (for adding transparency to unsegmented fibers), *Logic* (for logical operations between the ROIs), *Detect* (for a one-time segmentation execution), and *Export* (to save the segmented fibers). In this case of *Logic* option, the “0&1” operation is employed to identify fibers intersecting both sphere 0 (purple) and sphere 1 (green). **(C)** It shows the segmentation of the brain fibers that intersect the purple and green spheres while excluding those that pass through the blue sphere. In this case, we specified the logical operation (*Logic*) as “0&1&!2” within the *Extraction Tool* window.

For the query, different 3D objects check whether the node collides with or is inside the bounding box. In the first case, the algorithm continues recursively through the branch nodes until it reaches a leaf node, where the points are tested and added to the validator buffer if selected. In the latter case, all the points contained in the subnodes are translated into the corresponding fiber and marked as selected in the fiber validator buffer. The resulting selected fibers for each object can be used in logical mathematical operations (AND, OR, XOR, NOT). This allows for the use of multiple ROIs to find fibers connecting specific areas while excluding those selected by other areas. Figure 11 displays a selection of fibers that intersect two ROIs (green and purple), while excluding fibers that intersect the blue ROI.

## 3 Results

We executed the Phybers package on eight computers, each with different hardware and software configurations, as outlined in Table 3, listing features such as the CPU, graphics card, RAM, OpenGL version, OS, and Python version. The computers were sorted by CPU generation. To conduct the tests, we applied the following procedure: first, we installed Anaconda and created two virtual environments, one with Python 3.9 and another with Python 3.11. Subsequently, we installed the Phybers package from the repository using the command *$ pip install phybers*. Finally, we executed all the library commands to assess the different modules of the package (available as supplementary material). For each module execution, we randomly selected two subjects. One subject was sourced from the HCP database, while the second subject was derived from the ARCHI database. Additionally, a test was conducted in a Python 3.10 environment on the PC8 listed in Table 3.

**Table 3:** Provides an overview of the primary hardware and software characteristics evaluated while running the Phybers package, categorized by CPU generation.

**Computer**	**CPU**	**Graphics card**	**RAM**	**OpenGL Version**	**OS**	**Python Version**
**PC1**	Intel Core i9-12900	NVIDIA GeForce RTX 3060	128 GB	4.6.0	Ubuntu 22.04.1 LTS/ Windows 11	3.9 & 3.11
**PC2**	Intel Core i7-9700KF	NVIDIA Quadro P620	32 GB	4.6.0	Ubuntu 20.04.5 LTS	3.9 & 3.11
**PC3**	AMD Ryzen 9 5900HX	NVIDIA GeForce RTX 3060	24 GB	4.6.0	Windows 10	3.9 & 3.11
**PC4**	Intel Core i7 -8700K	NVIDIA GeForce GTX 1050 Ti	64 GB	4.6.0	Windows 10	3.9 & 3.11
**PC5**	Intel Core i7-7700HQ	Intel HD Graphics 630	64 GB	4.6.0	Ubuntu 20.04.2 LTS	3.9 & 3.11
**PC6**	Intel Core i7-12700K	NVIDIA GeForce GTX 1650	16GB	4.6.0	Ubuntu 22.04.2 LTS	3.9 & 3.11
**PC7**	Intel Core i5-8600K	NVIDIA GeForce GTX 1050 Ti	16 GB	3.0	Ubuntu 18.04.6 LTS	3.9 & 3.11
**PC8**	Intel Core i5-6600k	NVIDIA GTX 1660	16 GB	4.1.0	Windows 10	3.10

The first column assigns a unique number to each computer employed. Hardware resources are examined through the CPU, Graphics card, and RAM columns, whereas software resources are appraised via the columns for OpenGL version, OS (Operating System), and Python version. Phybers runs smoothly for all these listed features except for the Visualization module, which requires OpenGL versions higher than 3.0.

The installation of Phybers is straightforward via the *$ pip install phybers* command. The Segmentation, Clustering, and Utils modules function optimally across all the tested hardware and software configurations. Nonetheless, for the Visualization module, it is required to use OpenGL versions equal to or greater than 4.1.0, as earlier versions, such as 3.0 (PC7 in Table 3), lack support for certain functions. This hardware limitation extends to the graphics card, necessitating compatibility with OpenGL versions equal to or exceeding 4.1.0. Fortunately, OpenGL version 4.1.0 has been available since 2010, ensuring compatibility with graphics cards released thereafter. Regarding software prerequisites, Phybers offers compatibility with both Windows and Ubuntu systems. Users opting for MacOS are recommended to install a virtual machine. The recommended Windows versions include Windows 10 and Windows 11. Ubuntu users are encouraged to select from the following Long Term Support (LTS) versions: Ubuntu 18.04.6, Ubuntu 20.04.2, Ubuntu 20.04.5, Ubuntu 22.04.1, and Ubuntu 22.04.2. Lastly, Phybers seamlessly supports Python versions 3.9 and higher. Phybers' source code is publicly available on the GitHub repository. Additionally, it features a website that offers extensive and detailed documentation, along with examples and test data.

## 4 Discussion

In this study, we conducted the testing of the Phybers package on real neuroimaging data on eight computers with different configurations (Table 3). By conducting tests on computers with varying hardware and software configurations, we could fix some compatibility errors and ensure a comprehensive coverage of scenarios. This approach allowed us to identify potential strengths and weaknesses of the Phybers package, shedding light on its versatility and adaptability to different computing environments.

The Segmentation module enables fast segmentation of white matter fiber bundles from tractography dataset using a multi-subject atlas. The algorithm has been implemented with multicore processors and graphics processing units (GPUs), which allows for the segmentation of massive tractography datasets, and it has been tested with datasets containing up to 5.2 million fibers. To achieve this, the algorithm rapidly discards noisy fibers, leading to improved execution time and reduced memory usage (Vázquez et al., 2019). The algorithm allows a configurable threshold for each bundle in the atlas. The library provides three multi-subject atlases: one for DWM fibers (Guevara et al., 2012) and two for SWM fibers (Román et al., 2017, 2022). Additionally, any atlas of fibers in the MNI space with the specified format can be used. For example, we have segmented subjects from the HCP dataset using the atlas of long and short fibers from (Zhang et al., 2018). Segmentation results from this algorithm have been utilized in various clinical studies (Ji et al., 2019; Buyukturkoglu et al., 2022).

The Clustering module contains two exploratory fiber clustering algorithms that have proven their utility for analyzing fiber tractography datasets. These methods can be used as an initial exploration procedure to identify the main groups of fibers in a tractography dataset. Since the algorithms do not rely on anatomical data, they can be applied to any fiber configuration, as in Guevara et al. (2011) where an intra-subject clustering algorithm was applied to the FiberCup data (Poupon et al., 2008). Our library includes two fiber clustering methods: HClust (Román et al., 2017, 2022) and FFClust (Vázquez et al., 2020).

HClust is an automatic hierarchical method that can be applied to individual or multi-subject tractography dataset analysis. It is based on a distance metric between fibers and a threshold for dividing the dendrogram. The dendrogram is adaptively partitioned to get clusters with a maximum intra-clustering distance, a procedure that has proven to have a high power for disentangling WM fibers (Guevara et al., 2017, 2022; Román et al., 2017, 2022). However, it has a limitation on the number of input fibers due to its computation complexity and the calculation of all the pairwise fiber distances. This is the reason why we developed an FFClust fiber clustering algorithm designed for intra-subject clustering of massive tractography datasets.

FFClust is capable of capturing regular and compact clusters on a tractography dataset (Vázquez et al., 2020), on a reduced computation time (Vázquez et al., 2020), while obtaining high quality clusters, which was measured using the DB index (Davies and Bouldin, 1979). To deal with large datasets it uses several steps, based on the clustering of fiber points, following the principle that similar fibers will share the same point clusters. This algorithm was conceived as a first pre-processing step, hence it prefers to oversegment clusters than fuse groups of fibers with different shapes. As a limitation, it has a big set of input parameters, but for whole-brain tractography dataset many of them can be set to default values, and only a single value for both distance thresholds could require to be modified.

Users should evaluate which clustering method is more convenient, depending on their goal. Of course, results depend on the quality of tractography dataset and the registration method for the case of multi-subject analysis.

The Utils module provides a set of tools for tractography dataset analysis. Some of these tools are used internally by the other modules of the library, e.g., the tools for reading and saving fibers are used in all modules. However, we consider it necessary to provide the possibility to use these tools individually for any purpose of the user. The Deform sub-module allows the user to transform a tractography dataset to another space of a database providing a deformation image, such as the HCP database that provides the transformation to MNI space calculated with FSL software. Respecting the Sampling sub-module, both the segmentation and the clustering algorithms require that all fibers have the same number of points, which can be achieved using this module. The number of points depends on the application, and various numbers have been used, including 21 points (Guevara et al., 2012; Román et al., 2022), 12 points (Garyfallidis et al., 2012), 51 points (Garyfallidis et al., 2018), among others. The Intersection sub-module provides a similarity percentage of similarity between two fascicles that can be used to compare clustering or segmentation results. The Postprocessing sub-module generates a dataframe containing measurements from fiber sets (clusters or segmented), such as fiber bundle size, mean fiber bundle length (in *mm*), and intra-fiber bundle distance (in *mm*). These measurements enable the evaluation of both segmentation and clustering algorithms and facilitate filtering based on these features.

The Visualization module allows for the visualization of multiple objects in a single scene. This module enables the visualization of MRI images in NIfTI format, mesh data in *mesh* format, and brain tractography dataset in *bundles* and TRK formats. Various operations can be performed on each object, such as rotation, zoom, and panning. This module features a simple and user-friendly graphical interface. Furthermore, it provides a tool for the interactive segmentation of a set of brain fibers by placing two or more spherical ROIs. This tool is quite useful when exploring brain tractography dataset quickly and in real-time. It was implemented with an optimal use of OpenGL features to perform well on personal computers, and even some simplified components can execute on Mobile devices (Osorio et al., 2021). Several libraries developed for diffusion MRI data analysis include tools for data visualization. However, none of the software programs mentioned in Table 2 (Visualization column) have the feature to segment brain tractography dataset using 3D ROIs in real-time. On the other hand, our visualization software has the disadvantage of not being able to visualize diffusion MRI model glyphs, while packages such as SlicerDMRI, MRtriX, and Dipy have incorporated this tool.

In neuroscience, there is a wide variety of formats for tractography dataset files, MRI volumes, and meshes. The presented library has the limitation of supporting only a few input and output formats. It currently supports just four formats: *bundles* for tractography dataset, NIfTI for MRI, and *mesh* and GIfTI for meshes. In the state-of-the-art there are libraries that support other formats, for example: ExploreDTI, SliceDMRI, DSI Studio, and MRtrix. Future updates to our library may incorporate flexibility to read more formats or provide tools in the Utils module to convert among formats.

Respecting the library documentation, the choice of Sphinx as the primary tool for creating our documentation was based on several factors. Firstly, Sphinx offers remarkable ease of use and configuration, since its reStructuredText markup language is intuitive, enabling an efficient focus on content. Another notable advantage of Sphinx is its ability to generate documentation in multiple output formats, being selected HTML for our library. The inclusion of Sphinx in our development workflow played a significant role. The tool effortlessly fits into our current tools and processes, guaranteeing that the documentation remains up-to-date alongside source code changes. This ensures that users will always have access to the most recent information.

Finally, we packaged the library using the Python Package Index (*PyPI*), a widely used repository for software related to the Python programming language. This repository hosts a vast collection of projects, and facilitates easy installation of the library through the Python package manager (*pip*).

## 5 Conclusion

We propose a software library (Phybers) with state-of-the-art tools for analyzing brain fibers aiming to facilitate their use by the scientific community. It integrates tools such as fiber bundle segmentation, fiber clustering, and visualization algorithms that have been used separately in different studies. In addition, we integrated utility tools for sampling and transforming tractography datasets, calculating the intersection between fiber bundles and post-process brain fiber sets. The library provides sample data and extensive documentation. Furthermore, the library was developed with scalability in mind, therefore it is possible to integrate other existing state-of-the-art algoritmhs.

We believe that the generated library will facilitate the use of the included algorithms, achieving better sharing of state-of-the-art tools. As future work, we plan to integrate other methods such as the intersection of fibers with cortical meshes and a diffusion-based parcellation (López-López et al., 2020).

## Data availability statement

Publicly available datasets were analyzed in this study. This data can be found here: Phybers is freely available on a GitHub repository: https://github.com/phybers/phybers, under the GNU public license for non-commercial use and open-source development, which provides sample data. Extensive documentation is provided at: https://phybers.github.io/phybers.

## Ethics statement

Ethical approval was not required for the study involving humans in accordance with the local legislation and institutional requirements. Written informed consent to participate in this study was not required from the participants or the participants' legal guardians/next of kin in accordance with the national legislation and the institutional requirements.

## Author contributions

LG: Conceptualization, Methodology, Software, Writing—original draft, Writing—review & editing, Investigation, Visualization. IO: Software, Writing—review & editing. AC: Software, Writing—review & editing. HH: Investigation, Writing—original draft, Writing—review & editing. CR: Resources, Software, Writing—review & editing. CP: Writing—review & editing. J-FM: Writing—review & editing. CH: Conceptualization, Methodology, Supervision, Writing—original draft, Writing—review & editing. PG: Conceptualization, Funding acquisition, Methodology, Software, Supervision, Writing—original draft, Writing—review & editing.
